# Wolf Presence near a Temporary Sheep Pasture in Flanders: A Descriptive Camera-Trap Study

**DOI:** 10.3390/ani16040665

**Published:** 2026-02-19

**Authors:** Bert Driessen, Lore Pellens, Celine Bollen, Jasper Tavernier, Louis Freson

**Affiliations:** 1 Laboratory of Morphology, Department of Morphology, Medical Imaging, Orthopaedics, Physiotherapy and Nutrition, Faculty of Veterinary Medicine, Ghent University, 9820 Merelbeke, Belgium; 2 Animal Welfare Solutions, 3583 Paal, Belgium; 3 Federal Agency for Medicines and Health Products, 1210 Brussels, Belgium; 4 Agricultural Buildings Research, Department of Biosystems, KU Leuven, 3001 Heverlee, Belgium; 5 Laboratory of Humane Biology, Department of Physiology, KU Leuven, 3000 Leuven, Belgium

**Keywords:** wolves, livestock guardian dogs, electric fencing, human activity, recolonization

## Abstract

Wolves (*Canis lupus*) have recolonized Belgium after more than a century of absence, raising concerns about interactions with livestock in densely populated regions such as Flanders. Empirical field-based documentation of wolf behavior near protected livestock in such landscapes remains limited. This study presents a short-term, descriptive camera-trap case study documenting wolf presence near a temporary sheep pasture protected by electric fencing and livestock guardian dogs (LGDs). Nineteen camera traps monitored the pasture perimeter within a military training area in northeastern Flanders over a 16-day period in September 2023. Sheep were present for 11 days and accompanied by six LGDs. Twenty-three wolf images were recorded, corresponding to eight distinct detection events. Wolves were detected shortly after fence installation and following sheep removal. Occasional close approaches and fence inspection behavior were observed, but no fence crossings or predation events occurred. Most wolf detections occurred when sheep and LGDs were absent, although wolves were also recorded near periods of human activity. Given the observational design, causal inference is not possible. The study provides baseline documentation of wolf–livestock–LGD interactions in a densely populated European landscape.

## Simple Summary

The wolf (*Canis lupus*) has reappeared in Belgium after an absence of over a century, prompting concerns regarding interactions with both livestock and humans in a region encompassing a variety of agricultural, natural, and urban areas, such as Flanders. This study examined the possible interaction of wolves, sheep and livestock guardian dogs near a protected temporary pasture. Using motion-activated cameras over a period of sixteen days, we recorded wolves visiting the area several times, sometimes alone and sometimes in small groups of up to three animals. Most wolf detections occurred when sheep and dogs were absent. No fence crossings or predation events involving wolves were observed during the monitoring period. Interestingly, wolves were also observed during times of human and vehicle activity, indicating continued use of the area despite predictable human presence. Our observations highlight both the challenges and opportunities of coexistence. Rather than evaluating the effectiveness of protection measures, this study provides descriptive, site-specific documentation of wolf activity near a protected pasture in a densely populated landscape. These observations may serve as baseline information for future comparative and experimental research on wolf–livestock coexistence.

## 1. Introduction

Wolves (*Canis lupus*) have been recolonizing large parts of central and western Europe following the implementation of protection measures in the late 20th century [1,2]. After more than a century of absence, wolves returned to Belgium in 2018, when the first resident female (GW680f) established territory in Flanders [3,4]. Her identification was confirmed through established genetic monitoring protocols [4]. This recolonization has occurred in a human-dominated region, where livestock production, recreation, and other human activities are closely interwoven with remaining agricultural and natural areas. Subsequent to this initial recolonization, several wolves have settled or dispersed through the region, leading to the formation of resident packs [4]. As wolf presence increased, confirmed attacks on livestock have raised concerns among shepherds and land managers in the region [3,4].

Mitigation measures such as electric fencing and livestock guardian dogs (LGDs) are widely promoted within policy and management frameworks as primary tools to reduce livestock depredation [5,6]. While their effectiveness varies depending on the regional context, maintenance, and implementation quality, numerous studies demonstrate that well-maintained electric fences and properly integrated LGDs can substantially reduce predation risk [5,7]. However, much of the existing empirical evidence originates from mountainous or sparsely populated rural regions. In contrast, wolf behavior in response to deterrents and the performance of LGDs may differ in human-dominated landscapes where wolves have limited historical exposure to livestock protection measures and where human activity is frequent and spatially heterogeneous [8].

Empirical documentation of wolf interactions with protected livestock in densely populated, lowland regions of northwestern Europe remains limited. Although telemetry studies provide valuable information on movement ecology and territory use, camera traps offer complementary insights into fine-scale behavioral interactions at specific sites, including approach patterns, fence inspection, guardian responses by LGDs, and temporal overlap with livestock and human activity [8]. In Belgium, where most confirmed depredation cases occur in unprotected pastures [3], baseline documentation may support future evaluation of mitigation measures under local field conditions.

This study documents wolf interaction with livestock, LGDs and human activity around a temporary sheep pasture protected by electric fencing and six LGDs at the Belgian Air Force shooting range in Houthalen-Helchteren, located within the territory of the resident Hechtel-Eksel pack [9]. Over a 16-day monitoring period in September 2023, camera traps were used to address three descriptive objectives:

1. Document wolf presence patterns: record the frequency, timing, and spatial distribution of wolf detections near a protected pasture.
2. Characterize behavior of wolves, livestock and LGDs: describe activities in proximity to the fence, including approach behavior, guarding responses and fence inspection.
3. Describe temporal relationships: examine temporal associations between wolf activity, livestock presence, LGD activity and human activity.

This study was designed as an observational documentation of wolf behavior under operational field conditions rather than as an experimental test of deterrent effectiveness. The absence of control sites (i.e., unprotected pastures with comparable monitoring), the relatively short monitoring period, and the presence of confounding factors preclude causal inference regarding the effectiveness of individual mitigation measures. Instead, the study provides the first baseline documentation of wolf–livestock–LGD interactions at a protected site in Flanders, contributing empirical evidence from densely populated European landscape and informing the design of future comparative and experimental research.

## 2. Materials and Methods

### 2.1. Location

Belgium (385 residents per km^2^) and particularly Flanders, the northern region of the country (504 residents per km^2^) are densely populated and highly urbanized areas [10]. Flanders is characterized by a fine-scale land-use mosaic in which residential development, agricultural land, and semi-natural habitats (heathland, woodland, and military training areas) are closely interwoven. As a result, spatial boundaries between intensively managed and more natural areas are often permeable, and wolf movements, livestock husbandry and human activities frequently occur in close proximity.

This high degree of land-use interspersion may influence both wolf behavior and the practical performance of livestock protection measures, particularly in lowland regions with high levels of human activity. In combination with the recent recolonization of wolves, these landscape characteristics raise important questions regarding the applicability and effectiveness of coexistence strategies developed primarily in less densely populated regions [2,11].

### 2.2. Study Area

**Figure 1:**
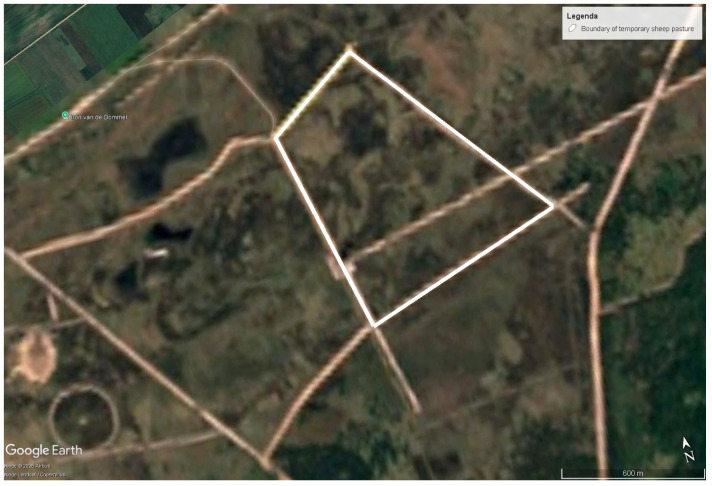
Satellite image of shooting range (the grazing pasture is delineated by white lines).

The study was conducted at the Belgian Air Force shooting range in Houthalen-Helchteren (Flanders, Belgium) between 4 and 19 September 2023. The military domain covers 2180 hectares and consists predominantly of open heathland interspersed with woodland and a network of sandy roads that provide high visibility for flight and training operations (Figure 1) [12,13]. The area supports extensive sheep grazing as a vegetation management tool to maintain heathland structure and limit fire risk.

Following the return of wolves to the region, sheep management practices at the site were modified: free-ranging grazing was replaced by temporary grazing pastures protected by electric fencing and LGDs. During the study period, sheep were confined within a temporary pasture established on heathland. The fenced enclosure covered approximately 0.7 km ^2^ and had a perimeter of 3.7 km. Sandy roads formed the external boundary of the enclosure, with the electric fence installed along the inner edge of these roads.

### 2.3. Electric Fencing

The pasture was enclosed using anti-wolf electric fencing installed in accordance with the guidelines of the Wolf Fencing Team [6]. The fence consisted of plastic and wooden poles supporting five electrified wires. The lowest wire was positioned less than 20 cm above ground level and the highest wire more than 120 cm above ground level. Wire spacing was approximately 20 cm for the lower wires and 30 cm for the upper wires.

Fence voltage was measured daily by the shepherd and a ranger during the period when sheep were present and consistently exceeded 4500 V, with typical values around 6300 V. After the removal of the sheep, fence voltage was no longer monitored and the exact timing of battery depletion is unknown. Consequently, interpretation of wolf behavior following livestock removal is limited. Vegetation along the sandy roads was sparse, minimizing the risk of grounding or short-circuiting of the electrified wires.

### 2.4. Camera-Trap Setup

A total of 19 camera traps were installed along the inner edge of the electric fence. The initial deployment on 4 September 2023 consisted of 13 cameras (Denver WCT-8020W (Denver, Hinnerup, Denmark) and Num’axes PIE1051 4G (Num’axes, Olivet, France)). Six additional Suntek HC-940Pro-Li cameras (Shenzhen Suntek Intelligent Technology Co., Shenzhen, China) were added on 15 September 2023. Technical specifications of the camera models are provided in Table 1.

Cameras were positioned 1.0–1.5 m inside the fence to maximize the detection of fence-approach behavior. Model selection reflected device availability and a trade-off between image resolution and 4G connectivity, which allowed for remote data retrieval. As a result, detection probabilities may have differed among cameras, and results are interpreted descriptively rather than comparatively.

Cameras were mounted on wooden poles at a height of approximately 1.5 m and oriented towards the sandy road and the area outside the fence.

**Figure 2:**
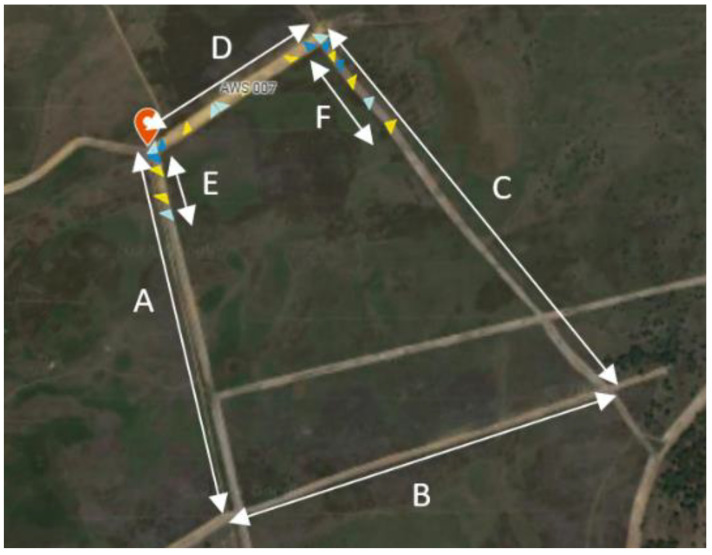
Satellite image of pasture with sides labelled (A–F). Cameras are placed alongside E, D and F (A: 875 m; B: 921 m; C: 1000 m; D: 625 m; E: 140 m; F: 240 m). Yellow: Denver, light blue: Suntek and darker blue: Numaxes.

Camera placement focused on sections of the pasture perimeter where wolf movements had been frequently observed prior to the study. Specifically, cameras monitored the shortest side of the pasture (625 m) and sections of two longer sides (140 m and 240 m; lines D, E, and F in Figure 2).

Each camera was programmed to capture a photograph followed immediately by a video segment upon activation. Photographs were classified as either containing visible activity (i.e., the presence of animals, humans, or vehicles) or no visible activity. Video recordings allowed for an additional outcome, namely the presence of auditory cues in the absence of visible subjects within the camera’s field of view. Such audio-only detections were retained and recorded during data processing, as they may indicate animal presence or activity occurring outside the visual range of the camera. Audio cues (e.g., sheep or livestock guardian dog vocalizations, electric fence sounds) were therefore systematically logged alongside visual detections.

### 2.5. Livestock and Livestock Guardian Dogs

A flock of 764 ewes and 6 rams (Flemish Sheep breed) grazed within the fenced pasture from 5 to 15 September 2023. Animal ages ranged from 19 months to 9 years. Typical body weights were approximately 75 kg for ewes and 90 kg for rams, with corresponding wither heights of 73 cm and 78 cm, respectively [14].

Six Spanish Mastiffs were used as LGDs. The dogs (three males and three females), aged 3–10 years, remained continuously with the flock throughout the grazing period. All dogs were neutered or spayed. Spanish Mastiffs typically reach wither heights of 72–77 cm [15].

### 2.6. Wolf Population

The study area lies within the territory of the Hechtel-Eksel pack. In 2023, the pack consisted of the breeding female GW1479f and her pups [3]. The resident male (GW979m) was killed in July 2023, prior to the study period [16]. At the time of data collection, the pups were approximately three months old. During this period, GW1479f remained resident in the area with her four surviving pups.

In the weeks following the death of the resident male, depredation incidents involving two sheep and one pony were confirmed within the pack territory [3].

Genetic identification of individual wolves was not conducted during this study. Consequently, all detections were classified at the species level (“wolf”) without assignment to individual animals.

### 2.7. Data Processing and Definitions

#### 2.7.1. Data Processing

A total of 3448 photographs and video recordings were reviewed and analyzed. Each file was annotated with a timestamp, the camera ID, the presence or absence of the observation categories listed in Table 2, and associated weather and light conditions. Data were recorded and organized in a Microsoft Excel spreadsheet.

Data analysis was conducted using R statistical software (version 4.3.1, 2023) to calculate frequencies and percentages, generate descriptive tables and to create visualizations [17]. No inferential statistical tests were applied, consistent with the descriptive nature of the study.

#### 2.7.2. Detection Event Classification

For analytical purposes, a distinction was made between individual images (each photograph or video file) and detection events. A detection event was defined as one or more images of the same species recorded within a 10-min interval at the same camera location. This conservative temporal clustering was applied to reduce pseudo-replication while acknowledging that repeated visits by the same individual, or the presence of multiple individuals, could occur within short time intervals.

Using this approach, 23 wolf images corresponded to eight distinct detection events. Both image counts and detection event counts are reported, as they provide complementary perspectives on wolf presence and activity patterns. This classification approach does not allow definitive distinction between repeated visits by the same individual, simultaneous presence of multiple individuals, or prolonged presence of the same individual with intermittent camera triggering.

## 3. Results

### 3.1. Monitoring Devices

#### 3.1.1. Camera Performance and Image Characteristics

**Figure 3:**
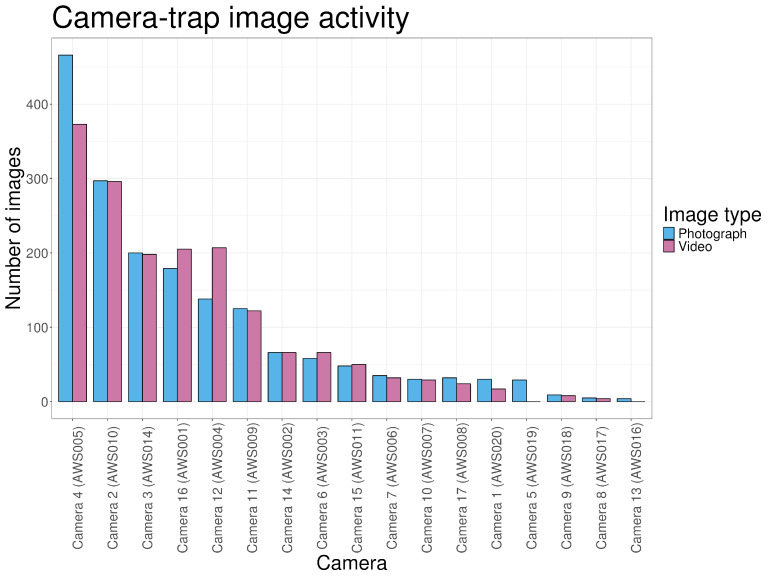
Number of images captured by each camera, sorted from the most active to the least active device.

Across the 16-day monitoring period, a total of 3448 images were obtained, consisting of 1751 photographs and 1697 videos. This is equivalent to approximately 380 h of video footage. Image output varied substantially among cameras (Figure 3). Cameras AWS016 and AWS019 recorded photographs only, while AWS015 and AWS012 captured no images during their deployment. Camera AWS005 recorded the highest number of images.

Of all images, 89.2% followed the intended photograph–video sequence. A further 6.21% consisted of photographs without an associated video, and 4.64% consisted of videos without a preceding photograph. On average, 85.1% of each camera’s output consisted of photographs, with photo-to-video ratios ranging from 0 to 1.50 among devices. Cameras AWS016 and AWS019 did not record any videos during the study period. In contrast, cameras AWS001, AWS003, AWS004 and AWS011 recorded proportionally more videos than photographs.

A total of 1377 images (39.9%) contained no visible animals, humans, or vehicles. Of these, 664 were videos in which audio signals (e.g., sheep bleating) indicated activity outside the camera’s field of view. No wolf vocalizations were recorded.

#### 3.1.2. Audio

With one exception, all video recordings included audio. The most frequently recorded sounds were electric fence ticking (N = 924; 54.5%), sheep vocalizations (N = 527; 31.1%) and LGD vocalizations (N = 84; 4.95%). Sheep vocalizations coincided with visible sheep in 311 videos (59.0%). A recurring unidentified beeping sound was recorded in 468 videos (27.6%). Additional sounds included passing aircraft, horses, human voices, and mobile phone ringtones (N = 375; 22.1%).

**Figure 4:**
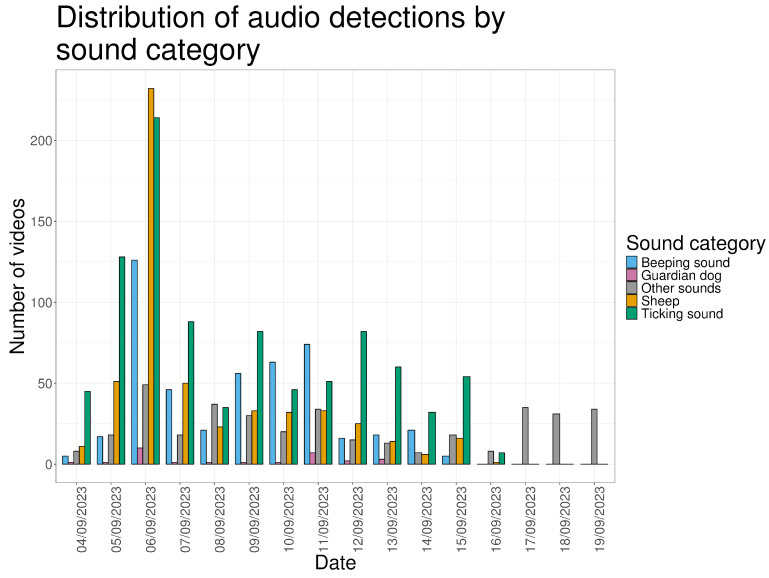
Number of videos containing audio grouped by the audio type (beeping sound, LGDs, sheep, ticking sound or other sounds).

An exceptional peak in sheep and LGD vocal activity occurred on 6 September, the day following the introduction of the flock into the pasture (Figure 4).

### 3.2. Weather Conditions

Most images were recorded under sunny conditions (N = 2305; 66.9%), followed by cloudy conditions (N = 734; 21.3%) and fog or mist affecting the lens (N = 140; 4.06%). Only three images were recorded during rainfall. Ambient temperatures during the study period ranged from 6.37 °C to 32.5 °C.

Wolf detections occurred under overcast conditions in 21.7% of cases and at temperatures ranging from 12.8 °C to 18.6 °C. Sheep and LGD detections occurred most frequently during sunny conditions.

### 3.3. Light Conditions

**Figure 5:**
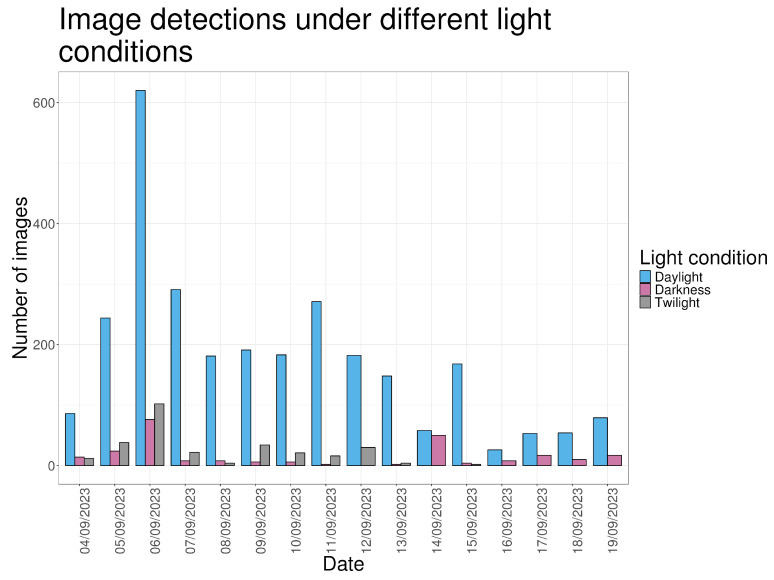
Number of images made during the study period grouped by the light condition.

Most images were captured during daylight (N = 2835; 82.2%). Twilight accounted for 285 images (8.27%) and darkness for 252 images (7.31%). A small proportion of images (N = 76; 2.20%) could not be classified due to condensation or technical artefacts (Figure 5).

Wolf detections occurred primarily during darkness (N = 15; 65.2%), with the remaining detections occurring during daylight (N = 8; 34.8%). No wolf detections were recorded during twilight. Sheep and LGDs were detected mainly during daylight hours (sheep: 86.6%; dogs: 63.3%).

### 3.4. Animals

#### 3.4.1. Wolves

**Figure 6:**
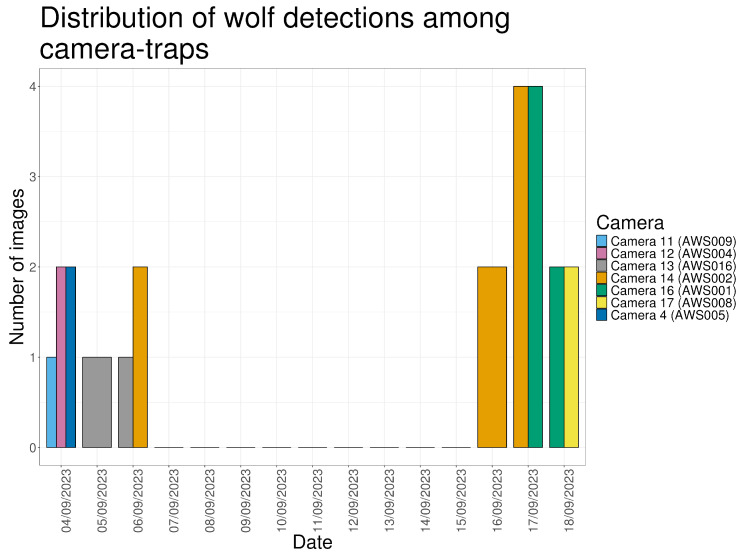
Number of images made by the cameras that captured at least one wolf during the study period.

Wolves were recorded in 23 images across six days of the monitoring period. These detections occurred during two distinct periods: 4–6 September (when sheep were present) and 16–18 September (after sheep removal). Eight detections included both photographs and videos, while seven detections consisted of a single media type (Figure 6).

**Figure 7:**
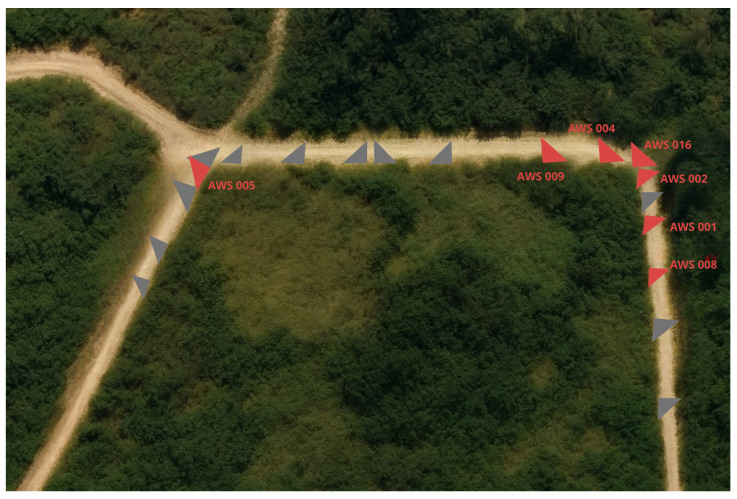
Satellite image contrast-enhanced and annotated for clarity using—standard image-editing software (GIMP, version 3.0.8); spatial content was not altered [18]. Red triangles indicate cameras that captured at least one instance of wolf activity; gray triangles indicate cameras with no wolf activity.

Wolf detections were most frequently recorded by cameras AWS002 and AWS001. Spatially, detections were concentrated near one corner of the pasture, while a single detection on 4 September 2023 occurred on the opposite side of the enclosure (Figure 7).

Wolf activity was recorded most frequently between 06:00–10:00 (N = 13; 56.5%) and between 21:00 and 23:00 (N = 8; 34.8%). One detection occurred at approximately 04:00, and one occurred during the afternoon (14:00).

**Figure 8:**
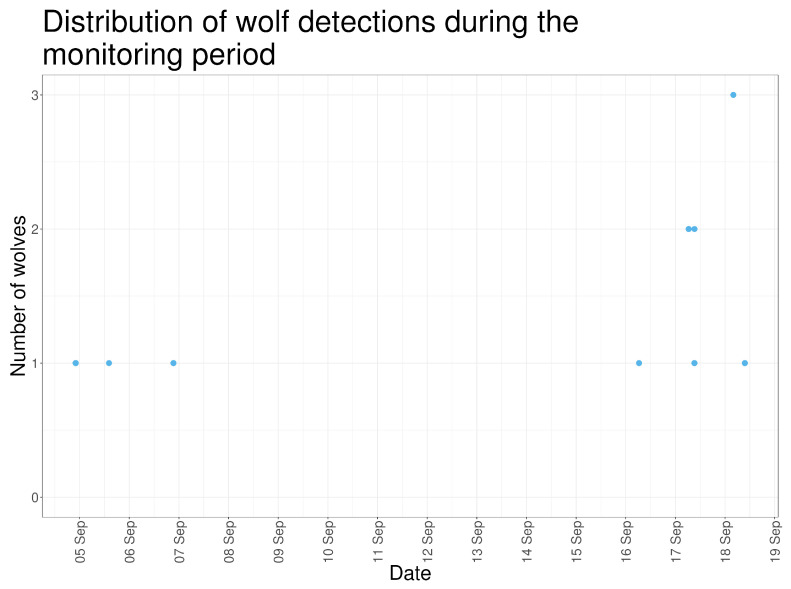
Temporal pattern of wolf detections across the entire monitoring period, displayed on a continuous time scale.

Most detections involved a single wolf (N = 17; 73.9%). Two wolves were recorded simultaneously in five images (21.7%), and three wolves were recorded in one image at 04:00 on 18 September 2023 (Figure 8).

In most detections, wolves were classified as walking or trotting (N = 17; 73.9%). Behavior classified as fence inspection was recorded in three detections (13.0%) by cameras AWS002 and AWS001. During these detections, wolves approached within approximately two meters of the fence, and all occurred during darkness. On 6 September 2023, these detections coincided with recordings that included sheep vocalizations. No entry attempts were recorded, and no predation events occurred during the study period.

#### 3.4.2. Livestock and Livestock Guardian Dogs

**Figure 9:**
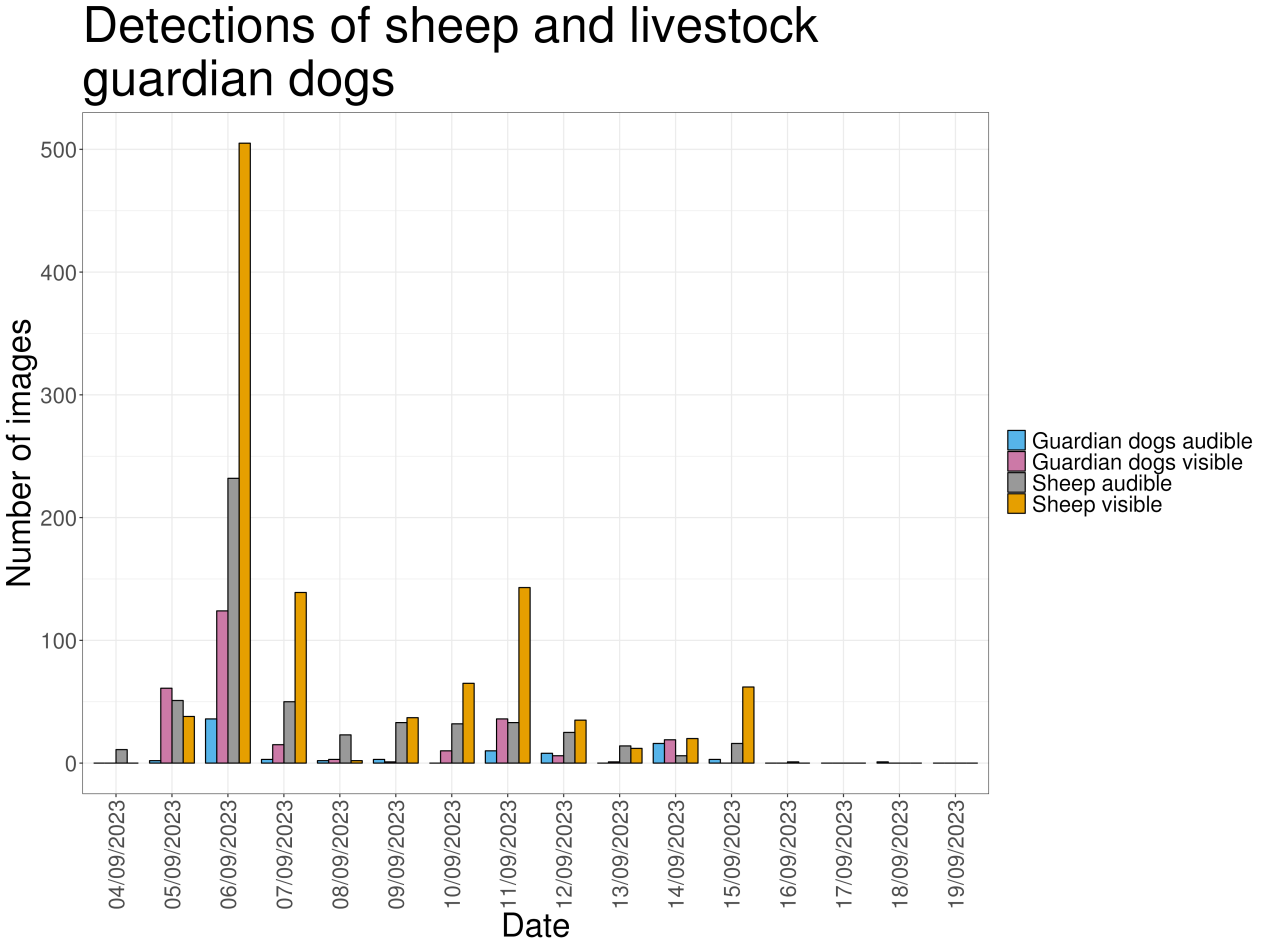
The number of images in which sheep and LGDs are visible an audible, for every day in the study period.

Sheep were recorded in 1058 images (30.7%), with detections occurring daily between 7:30 and 21:00 while the flock was present in the pasture (5–15 September 2023). Sheep detections peaked on 6 September 2023 (Figure 9). Recorded sheep behaviors primarily included grazing or walking.

LGDs were recorded in 276 images (8.0%). Most detections occurred during daylight (63.3%), followed by darkness (23.3%) and twilight (13.5%). LGDs were recorded most alone 77.9% of the images, with two dogs present in 17.8% and three dogs in 3.6%. Behavior classified as active guarding was recorded in 47 images (17.0%), predominantly during darkness (89.4%). Barking was recorded in 83 images (2.41%), mostly during darkness. In 41% of these recordings, a dog was visible in the frame. Actively guarding behavior and sheep presence were recorded simultaneously in 6.38% of the images.

#### 3.4.3. Other Wildlife

**Figure 10:**
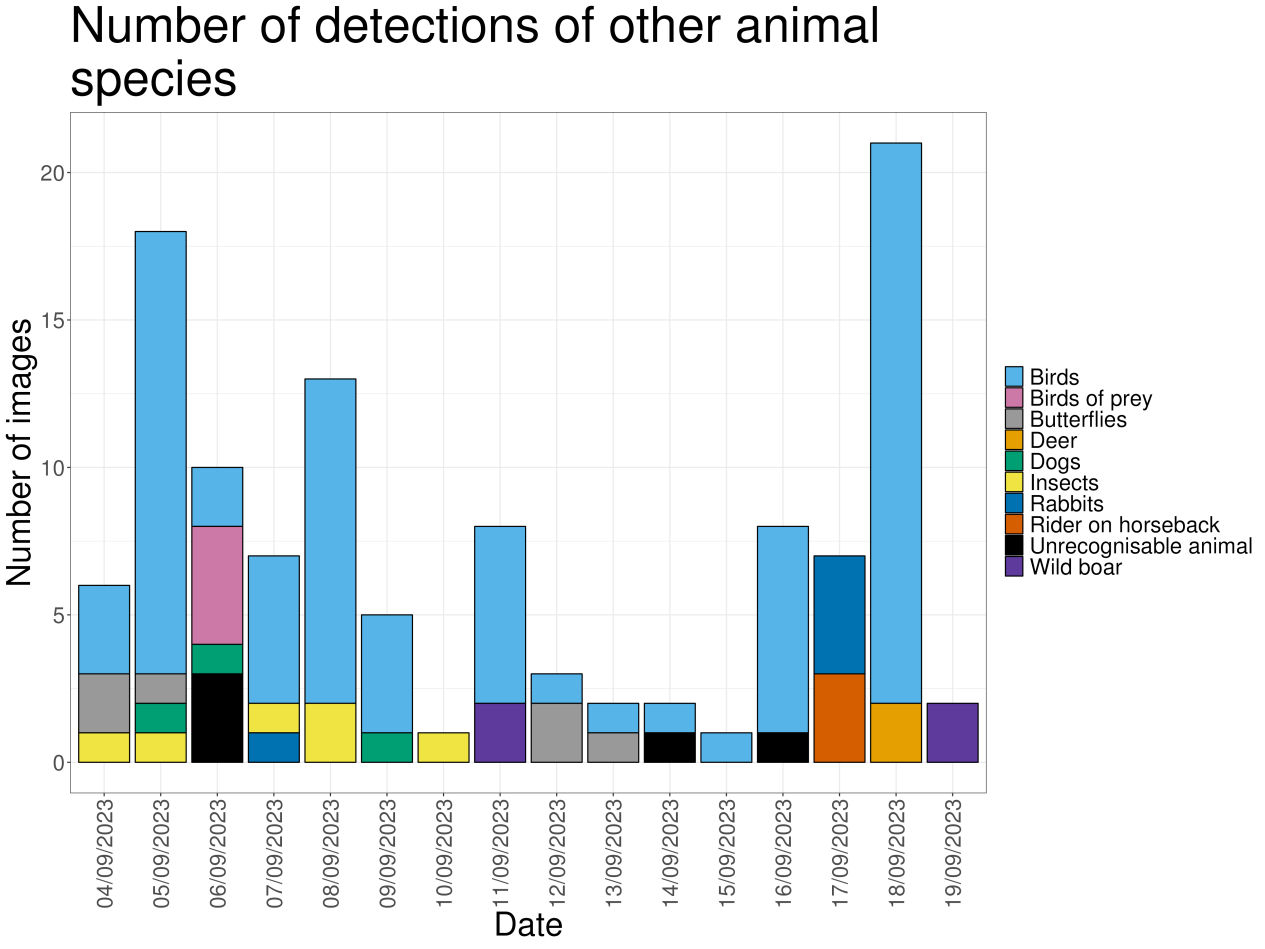
The number of images in which other types of animals are visible, for every day in the study period.

Detections of non-target wildlife species are reported to document baseline animal activity, confirm camera performance, and provide contextual information relevant to interpreting wolf behavior. A total of 114 detections of other wildlife species were recorded, including birds (70.2%), insects (5.3%), rabbits (4.4%), wild boar (3.5%), and deer (1.75%) (Figure 10). Most detections occurred during daylight (83.2%). One deer was recorded jumping through the fence on 18 September, likely after the electric current had ceased.

**Figure 11:**
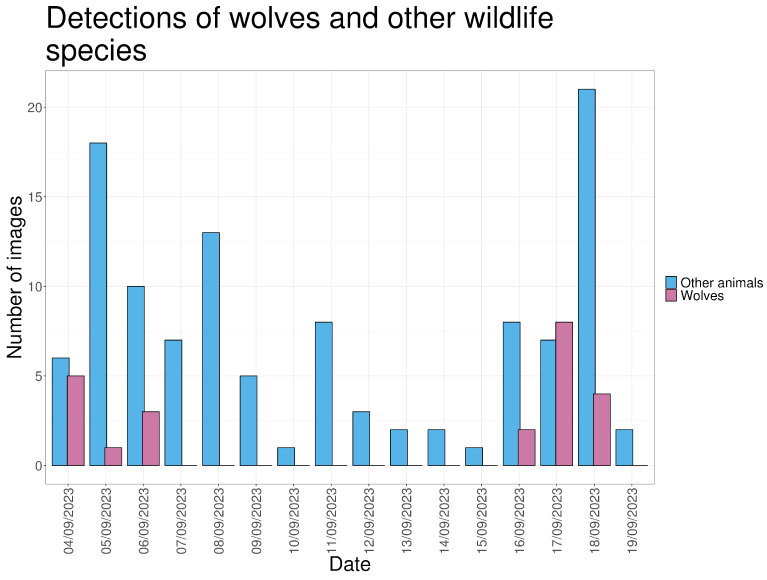
The number of images in which wolves and other wildlife animal species are visible, for every day in the study period.

On average, approximately seven detections of other wildlife species were recorded per day. During days when wolf detections occurred, the number of wildlife detections was higher (11–12 detections per day) than on days without wolf detections (Figure 11).

### 3.5. Human Interventions

#### 3.5.1. Vehicles

**Figure 12:**
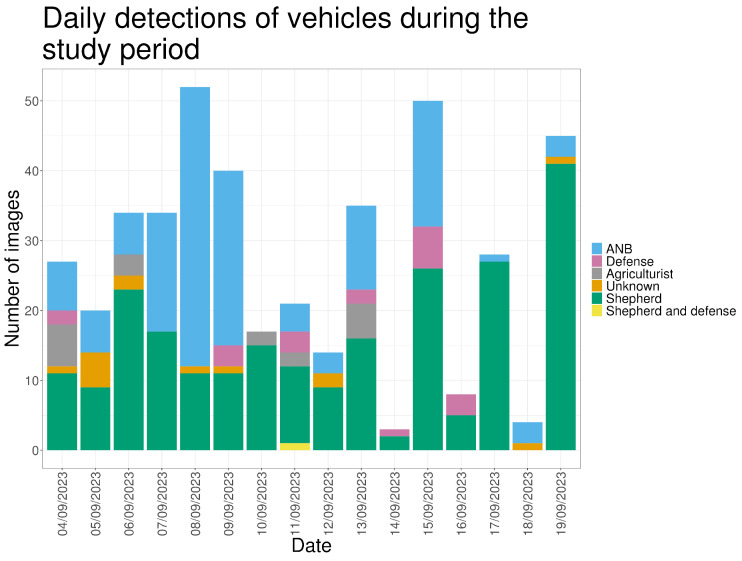
Daily detections of vehicles during the monitoring period.

Vehicle detections were used as an indicator of human activity and operational disturbance within the study area, rather than as a focal variable. Vehicles were recorded in 432 images (12.5%), predominantly during daylight (93.8%). The shepherd, either alone or accompanied, accounted for 54.4% of vehicle detections, while staff from the Agency for Nature and Forests (ANB) accounted for 30.6% (Figure 12).

**Figure 13:**
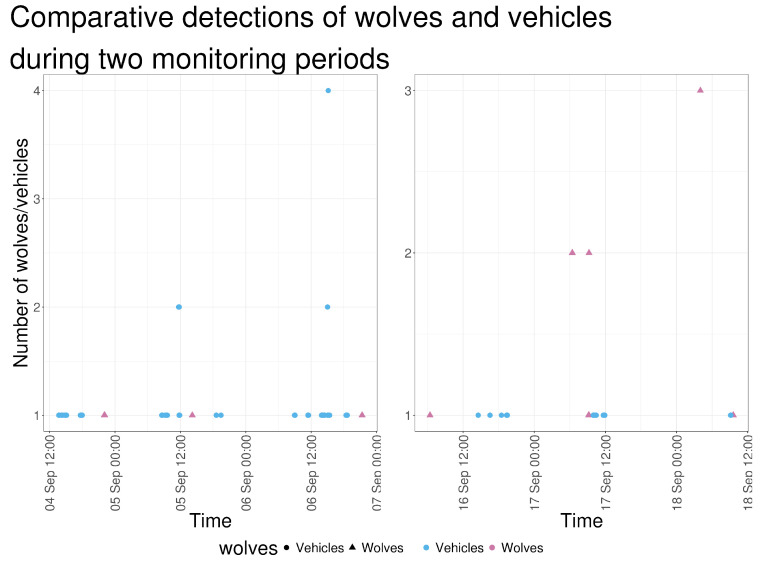
Number of wolves and vehicles detected in camera-trap images along a continuous timeline covering the two periods of wolf presence (4–6 September 2023 and 16–18 September 2023).

Vehicle detections occurred regularly throughout the monitoring period and at various times of the day. Vehicle activity was also recorded during both periods in which wolves were detected (Figure 13).

#### 3.5.2. Pedestrians

**Figure 14:**
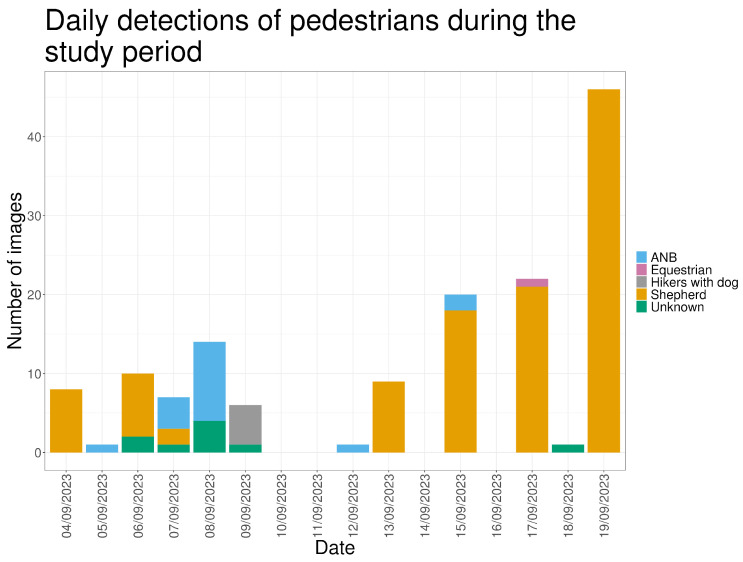
The number of images in which pedestrians are visible, for every day in the study period.

Pedestrians were recorded in 145 images (4.21%), almost exclusively during daylight hours (98.6%) (Figure 14). The shepherd, either alone or accompanied, accounted for 77.2% of pedestrian detections.

**Figure 15:**
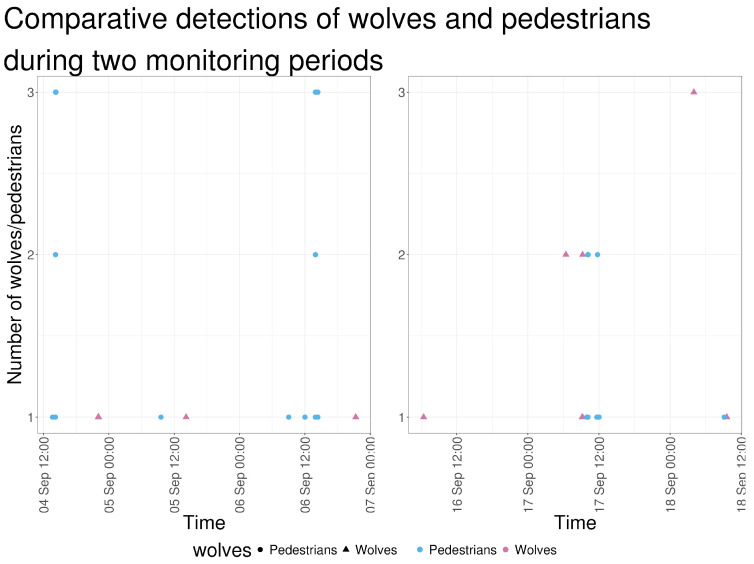
Number of wolves and pedestrians detected in camera-trap images along a continuous timeline covering the two periods of wolf presence (4–6 September 2023 and 16–18 September 2023).

On two occasions, wolf detections were recorded within approximately 30–45 min of pedestrian and vehicle activity (Figure 15). On 17 September 2023, wolves were detected at 09:09 and 09:13, followed by the shepherd’s vehicle at 09:56 and a shepherd’s worker on foot shortly thereafter. On 18 September 2023, wolves were detected at 09:34 and 09:32; earlier that morning, an ANB vehicle (09:06) and a pedestrian identified as an ANB staff member (09:07) were recorded. These detections were recorded by cameras located on different sections of the pasture perimeter.

### 3.6. Overview

**Figure 16:**
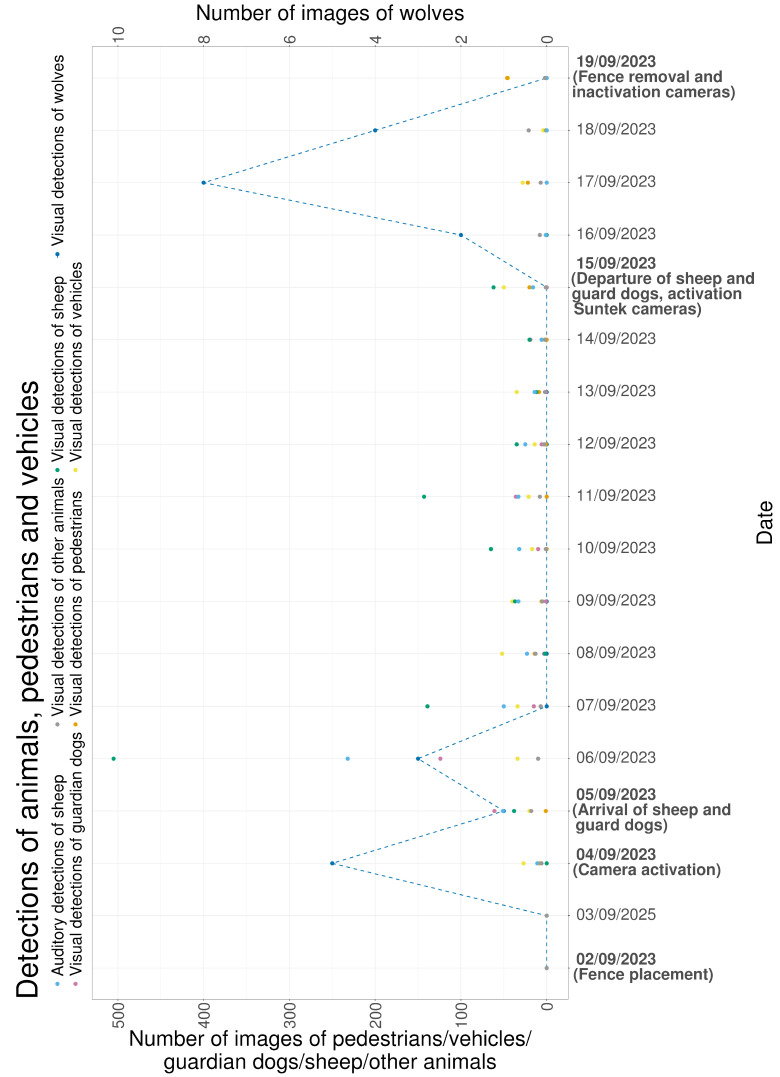
Overview of the number of images containing sheep, LGDs, wolves, other wildlife species, pedestrians and vehicles.

Figure 16 summarizes detections across the full monitoring period. Wolf detections occurred during the first three days of monitoring and during the three days following sheep removal. Sheep detections increased on 15 September 2023, the final day of grazing in the pasture. LGD detections followed a similar temporal pattern to sheep detections.

**Figure 17:**
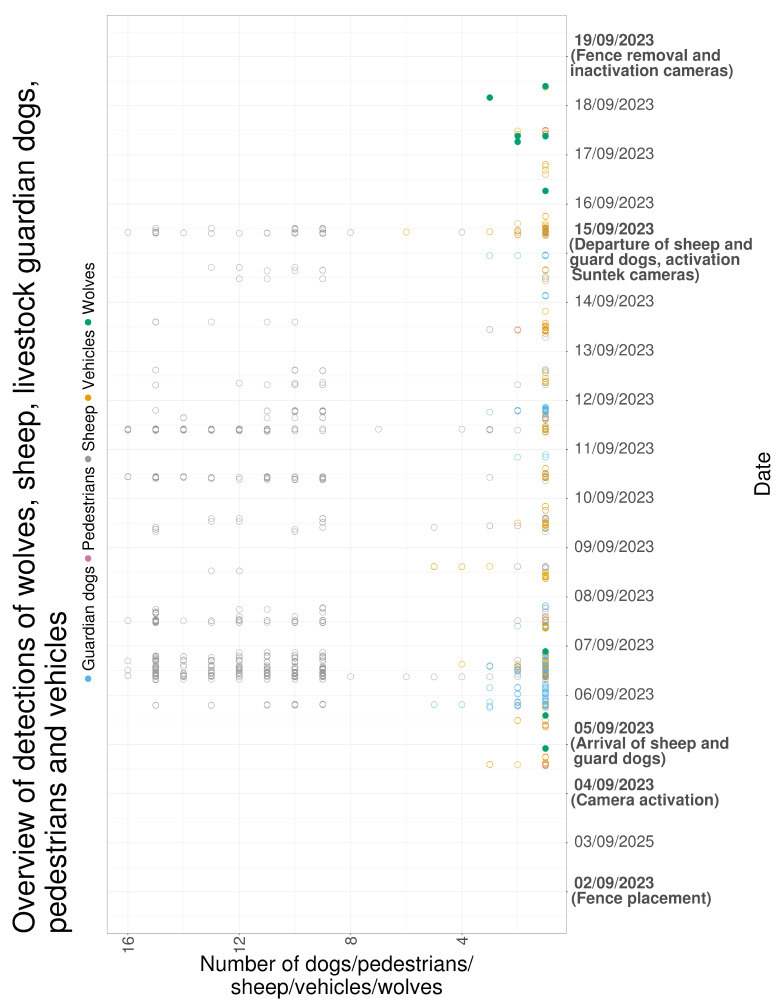
Overview of the number of sheep, LGDs, wolves, pedestrians and vehicles captured by the cameras on a continuous timescale.

Figure 17 presents a continuous timeline of detections. Periods with high sheep and LGD activity, particularly on 6 September 2023, were followed by wolf detections later the same day. After livestock removal on 15 September 2023, wolf detections were recorded for three consecutive days.

## 4. Discussion

This study provides a short-term, observational case study of wolf activity around a protected sheep pasture in a densely human-used landscape. The limited number of wolf detections constrains inference and precludes causal conclusions; results are therefore interpreted descriptively and in relation to existing literature rather than as definitive tests of protection effectiveness.

### 4.1. Wolf Behavior in Relation to the Pasture and Protective Measures

Wolf detections were clustered into distinct temporal periods that coincided with changes in livestock management, namely the installation of fencing and the subsequent removal of sheep. This pattern may indicate that wolves continued to use the area irrespective of livestock presence, although alternative explanations cannot be excluded. Similar dynamic responses to livestock availability and husbandry practices have been documented elsewhere, indicating that wolves rapidly adjust space use in response to changing risk–reward landscapes [8].

Most detections involved single individuals, although small groups were occasionally recorded. Solitary detections do not necessarily indicate dispersing wolves, as pack members may travel independently during routine territorial movements [19,20].

The study cameras together with ANB camera traps, wolf tracks and scat observations, suggest that wolves consistently approached the pasture from the same direction, indicating the use of habitual travel routes [21]. Repeated use of fixed paths is well documented in wolves and may reflect terrain features that facilitate movement, established scent-marking routes, or predictable access points such as sandy roads. From a management perspective, identifying such recurrent approach zones is relevant because it allows targeted reinforcement of deterrents rather than uniform intensification along the entire perimeter.

Wolf detections occurred predominantly during early morning and late evening hours, consistent with established circadian activity patterns in European wolves [22]. Although detections coincided with a relatively narrow temperature range, this likely reflects the timing of activity rather than thermal preferences per se.

The absence of fence crossings observed in this study is consistent with previous reports indicating that combinations of electric fencing and LGDs may increase perceived risk for wolves, although causal inference is not possible here. Contemporaneous wolf attacks on unprotected flocks in the surrounding region provide broader regional context but cannot be directly attributed to the conditions observed at the monitored pasture [3].

### 4.2. Livestock, Livestock Guardian Dogs and Their Responses

Sheep and LGDs showed elevated activity shortly after introduction to the pasture, a pattern that may reflect both acclimatisation to a new environment and heightened responsiveness to perceived threats. Increased livestock movement has been observed in other systems during predator presence, including sensor-based studies demonstrating elevated activity levels prior to predator detection [23]. However, adjustment effects unrelated to predation risk are also well documented [24], and both explanations remain plausible in this case.

LGD behavior was characterized by increased nighttime activity and guarding responses, consistent with their role in deterring nocturnal predators [25]. The limited spatial overlap between actively guarding LGDs and sheep suggests that dogs moved toward perceived threats rather than remaining within the flock, a behavior expected of experienced guardian dogs. Wolves approached the fence but did not attempt entry, indicating that the combined sensory cues associated with LGDs and electric fencing may have elevated perceived risk beyond acceptable thresholds.

### 4.3. Human Activity and Wolf Responses

The study area was characterized by frequent and diverse human activity, including vehicles, pedestrians, and military operations. Wolves nevertheless continued to use the area and were detected within short temporal windows following human presence. This limited temporal avoidance aligns with evidence that wolf responses to humans vary with local habituation, predictability of disturbance, and perceived lethality [26].

The use of an active military domain by wolves mirrors patterns reported from Germany and other recolonized regions, where wolves frequently establish territories in military training areas [27]. Such areas combine extensive semi-natural habitat with predictable, non-lethal human activity and reduced risk of persecution, supporting coexistence despite regular disturbance.

### 4.4. Contextualizing Livestock Protection Effectiveness

During the study period, genetically confirmed wolf attacks occurred on unprotected flocks within the broader region [3], while no predation occurred at the protected pasture monitored here. Although this contrast is suggestive, the observational nature of the study prevents definitive attribution of outcomes to specific protective measures.

Multiple, non-exclusive factors may explain the observed pattern. First, spatial positioning within the territory may have influenced encounter rates independently of protection, with wolves encountering unprotected flocks earlier along habitual routes. Second, individual variation in wolf behavior and risk tolerance may limit generalization, as responses by a single pack or individual do not necessarily reflect broader population-level behavior. Third, cumulative risk perception arising from LGDs, electric fencing, and regular human activity may have rendered the pasture unattractive relative to alternative prey sources.

Despite these uncertainties, the observations align with a growing body of European evidence supporting the use of layered protection strategies to reduce wolf–livestock conflict [5,7,28]. The observations are consistent with existing evidence supporting the application of combined measures in densely populated regions such as Flanders, while underscoring the need for longer-term, replicated studies to disentangle the relative contributions of individual deterrents and to assess potential behavioral adaptation by wolves over time.

## 5. Conclusions

This observational study provides the first camera trap-based documentation of wolf behavior in the immediate vicinity of livestock protected by a combination of electric fencing and LGDs in Flanders, Belgium. Despite repeated wolf presence near the pasture during a 16-day monitoring period, no predation occurred during the 11 days in which sheep occupied the enclosure. These observations document site-specific wolf activity patterns near a protected pasture but do not allow causal inference regarding the effectiveness of individual protection measures.

Wolves showed limited temporal avoidance of predictable human activity, with detections occurring within 30–45 min of pedestrian and vehicle presence. They consistently approached the pasture from the same direction and occasionally inspected the fence at close range, yet no entry attempts were observed. These behavioral patterns document continued use of the area under the monitored conditions, while responses to deterrents cannot be attributed to specific mechanisms within the present study design.

While the limited number of wolf detections and the short monitoring period preclude causal inference, the study provides important baseline information for a region where empirical data on wolf–livestock interactions remain scarce. Rather than evaluating livestock protection effectiveness, the study offers descriptive documentation of wolf–livestock–LGD interactions in a densely populated, human-modified landscape. The observations are consistent with findings from other European systems reporting reduced depredation at protected sites. However, such consistency should be interpreted as contextual rather than as evidence derived from experimental testing.

Future research should prioritize replicated, multi-site comparisons of protected and unprotected pastures; systematic variation of protective elements; integration of GPS telemetry where feasible; and long-term monitoring of wolf behavioral adaptation to robustly assess livestock protection effectiveness.

## Figures and Tables

**Table 1:** Camera types, names and characteristics.

Cameras	Camera Type	Manufacturer	Source City, Country	Type	Distance IR	Angle Lens	Angle PIR	Resolution	Type IR
AWS 001, AWS 002, AWS 003, AWS 004 and AWS 005	Num’axes PIE1051 4G	Num’axes	Olivet, France	4G	20 m	100°	100°	24MP	940 nm
AWS 006, AWS 007, AWS 008, AWS 009, AWS 010, AWS 011, AWS 012 and AWS 014	Denver WCT- 8020W	Denver	Hinnerup, Denmark	Wi-Fi	25 m	90°	120°	12MP	940 nm
AWS 015, AWS 016, AWS 017, AWS 018, AWS 019 and AWS 020	Suntek HC-940 Pro-Li	Suntek	Shenzhen, China	4G	30 m	90°	120°	36MP	940 nm

**Table 2:** Observations listed in Excel Spreadsheet.

Animal or Human Activity	Yes or No
Wolf	Number: 1, 2 or 3
	Time of activity: morning, afternoon, evening or night
	Activity: walking, trotting, observing the ground, observing the fence, …
	Location: next to fence, in tire track, behind tire track, close to or in the vegetation
	Direction: towards the camera, away from the camera or other
	Distance from the fence: close, a few metres from the fence or far
	Electricity on fence present: unknown, yes or no
	Sheep and dogs present in pasture: yes or no
Vehicle	Appearance vehicle: description
	Number: 1, 2, 3, 4 or multiple
	Owner: shepherd, military, ANB or unknown
	Direction: towards the camera, away from the camera or other
	Leaving vehicle: unknown, yes or no
Human	Who: shepherd, ANB, horseback rider, hikers or unknown
	Number: 1, 2, 3, 4, multiple or unknown
	Measuring electricity: unknown, yes or no
	Activity: installation camera, tearing down fence, feeding or looking after the dogs, observing the ground, taking pictures, walking, talking or other
Livestock guardian dog	Number: 1, 2, 3, 4 or multiple
	Actively guarding: yes or no
	Other activity: lying or sitting, walking or running, observing environment, sniffing, urinating, being around people or sitting in trailer
Sheep	Number: 1 to 17 or flock
	Activity: walking, grazing, ruminating, lying, observing environment, sniffing or other
Other	Other visual observations
Audio	Auditory observations
Light	Daylight, dark or twilight
Weather	Sunny, cloudy, rain, fog or unspecified

## Data Availability

Data are available on request from the corresponding author.
